# Endocrine Implications of Thyroid Incidentalomas Detected During Lymphoma Staging With 18F‐Fluorodeoxyglucose Positron Emission Tomography

**DOI:** 10.1111/cen.15295

**Published:** 2025-06-25

**Authors:** Marcos Tadashi Kakitani Toyoshima, Roberta Morgado Ferreira Zuppani, Isabelle Pinheiro Amaro, Camila Regina Pereira Batista de Macedo, Ricardo Miguel Costa de Freitas, Rodrigo Dolphini Velasques, Juliana Pereira, Rafael Loch Batista, Debora Lúcia Seguro Danilovic, Delmar Muniz Lourenço, Ana O. Hoff

**Affiliations:** ^1^ Endocrine Oncology Service, Instituto do Câncer do Estado de São Paulo Octávio Frias de Oliveira Hospital das Clínicas da Faculdade de Medicina da Universidade de São Paulo São Paulo Brazil; ^2^ Department of Radiology and Oncology Hospital das Clínicas da Faculdade de Medicina da Universidade de São Paulo São Paulo Brazil; ^3^ Department of Internal Medicine, Division of Endocrinology and Metabolism Hospital das Clínicas da Faculdade de Medicina da Universidade de São Paulo São Paulo Brazil; ^4^ Division of Hematology, Transfusion Medicine and Cell Therapy Hospital das Clínicas da Faculdade de Medicina da Universidade de São Paulo São Paulo Brazil; ^5^ Laboratorio de Endocrinologia Celular e Molecular (LIM 25) Hospital das Clínicas da Faculdade de Medicina da Universidade de Sao Paulo São Paulo Brazil

**Keywords:** FDG‐PET, lymphoma, thyroid autoimmunity, Thyroid incidentalomas, thyroid malignancy

## Abstract

**Objective:**

This study investigated the prevalence, characteristics, and endocrine implications of thyroid incidentalomas detected during lymphoma staging using FDG‐PET/CT.

**Design:**

Retrospective cohort study.

**Patients:**

A total of 795 adult patients with lymphoma who underwent FDG‐PET/CT for staging at a tertiary oncology centre were included.

**Measurements:**

Thyroid uptake was classified as focal, diffuse, or absent. Additional data included demographic features, lymphoma subtype, thyroid function, autoantibodies, ultrasound (US), fine‐needle aspiration cytology (FNAC), and histopathology when available.

**Results:**

Thyroid abnormalities were detected in 139 patients (17.5%). Focal FDG uptake was observed in 33 patients (4.2%) and was associated with a malignancy rate of 18.2% (28.6% among those who underwent FNAC). Diffuse uptake was observed in 20 patients (2.5%) and was significantly associated with positive thyroid autoantibodies (58.3%, *p* < 0.01). Older age and female gender were independent predictors of thyroid uptake. ROC analysis identified optimal age thresholds of 68.6 years for females and 59.8 years for males (AUC = 0.72). SUVmax, Hounsfield units, and ACR TI‐RADS classification were not significantly associated with malignancy. All malignant cases occurred in nodules classified as TI‐RADS 4 or 5. Volume on CT was inversely associated with malignancy (*ρ* = –0.50, *p* = 0.046). No significant impact on overall survival was observed.

**Conclusions:**

Thyroid incidentalomas are frequent during lymphoma staging by FDG‐PET/CT and should be appropriately evaluated. Focal uptake carries a relevant malignancy risk, even in nodules with low SUVmax. Diffuse uptake often reflects autoimmune thyroiditis. A multimodal diagnostic approach is essential to guide management and avoid unnecessary delays in cancer care.

## 1 Introduction

Haematolymphoid involvement in non‐haematopoietic tissues, including endocrine organs, poses significant diagnostic challenges, particularly when lymphoma secondarily affects the thyroid gland [1]. Positron emission tomography combined with 18F‐fluorodeoxyglucose (FDG‐PET/CT) has become an essential tool in the management of lymphoma, contributing to staging, monitoring therapeutic responses, and prognostic evaluation [2, 3, 4].

Incidental thyroid uptake detected on FDG‐PET/CT frequently raises concerns regarding potential malignancy or underlying benign thyroid disorders [5, 6, 7, 8]. The reported prevalence of thyroid incidentalomas identified on FDG‐PET varies from 1.2% to 4%, with malignancy rates reaching up to 35% among cases with focal uptake [9, 10].

In this context, understanding the prevalence and clinical characteristics of thyroid incidentalomas in lymphoma patients is crucial for ensuring accurate diagnosis and guiding optimal management strategies. Therefore, this study aims to analyse incidental thyroid findings detected during lymphoma staging with FDG‐PET/CT, with a focus on their association with demographic factors, lymphoma subtype, and clinical outcomes.

## 2 Materials and Methods

This retrospective cohort study analysed data from lymphoma patients who underwent FDG‐PET/CT scans for staging at our institution between January 2018 and October 2023. Inclusion criteria comprised patients aged 18 years or older with a confirmed diagnosis of lymphoma and available FDG‐PET/CT imaging data. Exclusion criteria included a history of thyroidectomy, non‐lymphoma‐related diagnoses, or incomplete imaging data.

Of the 900 patients initially reviewed, 22 were excluded due to alternative diagnoses (e.g. multiple myeloma, Erdheim‐Chester disease, Rosai‐Dorfman disease, Langerhans cell histiocytosis, histiocytic sarcoma, Castleman disease, blastic plasmacytoid dendritic cell neoplasm, and lymphoid hyperplasia), one due to prior thyroidectomy, and four were excluded because they were diagnosed with thyroid lymphomas presenting as cervical enlargement: these cases are detailed in a separate manuscript [11]. An additional 78 patients were excluded due to incomplete or unavailable FDG‐PET/CT data. The final cohort comprised 795 eligible patients. Patients were followed until their last available consultation, up to December 2024. Ethical approval for this study was obtained from the local ethics committee (CAAE: 74292323.8.0000.0068).

### 2.1 Data Collection and Imaging Review

Demographic data (age, gender), clinical data (lymphoma subtype, treatment history), and imaging findings were collected from electronic medical records. Thyroid uptake on FDG‐PET/CT was classified as focal, diffuse, or absent based on visual assessment by experienced nuclear medicine physicians blinded to clinical outcomes. Maximum standardized uptake values (SUVmax) were recorded for thyroid lesions when available. For the analysis of computed tomography (CT) features, volumetric data (in cm³) and Hounsfield unit (HU) measurements were extracted from non‐contrast CT scans. FDG‐PET/CT scans were reviewed independently by two nuclear medicine specialists to ensure consistency in thyroid uptake classification. Discrepancies were resolved by consensus. Thyroid function tests (thyroid‐stimulating hormone [TSH] and free thyroxine [T4]) and thyroid autoantibodies (thyroid peroxidase antibody and thyroglobulin antibody) were included when available. Additionally, thyroid ultrasound findings, ultrasound‐guided fine‐needle aspiration cytology (US‐guided FNAC), and histopathological results from thyroidectomy specimens were collected when indicated.

### 2.2 Statistical Analysis

Continuous variables were expressed as mean ± standard deviation (SD) or median with 25th and 75th percentiles (interquartile range—P25–P75), as appropriate. Comparisons between groups were performed using independent t‐tests or Mann‐Whitney U tests for continuous variables and chi‐square tests or Fisher's exact tests for categorical variables. Correlations between SUVmax values and ordinal variables (ACR‐TIRADS and Bethesda classifications) were assessed using Spearman's rank correlation coefficient.

Logistic regression analyses were conducted to identify independent predictors of thyroid uptake, including age, gender, and lymphoma subtype. Odds ratios (ORs) with 95% confidence intervals (CI) were reported. Receiver Operating Characteristic (ROC) curve analysis was used to determine optimal cut‐off values for continuous variables. For age, the optimal thresholds for predicting thyroid uptake were identified separately by gender, and for SUVmax, the best cut‐off to differentiate malignant from benign thyroid lesions was determined. In both analyses, Youden's J statistic was used to identify the optimal points, and sensitivity, specificity, and area under the curve (AUC) were reported.

Kaplan‐Meier survival analyses were performed to assess the impact of thyroid uptake on overall survival.

All tests were two‐tailed, and statistical significance was set at *p* < 0.05. Analyses were conducted using the R language (version 4.4.1, R Development Core Team) within the RStudio integrated development environment (version 2024.09.1 + 394, Posit Software, Boston, MA).

## 3 Results

The final cohort included 795 patients, with a mean age of 50.9  ±  17.9 years, and 366 were females (46.0%). Hodgkin lymphoma (HL) was diagnosed in 260 patients (32.7%), and non‐Hodgkin lymphoma (NHL) in 535 patients (67.3%).

**Figure 1:**
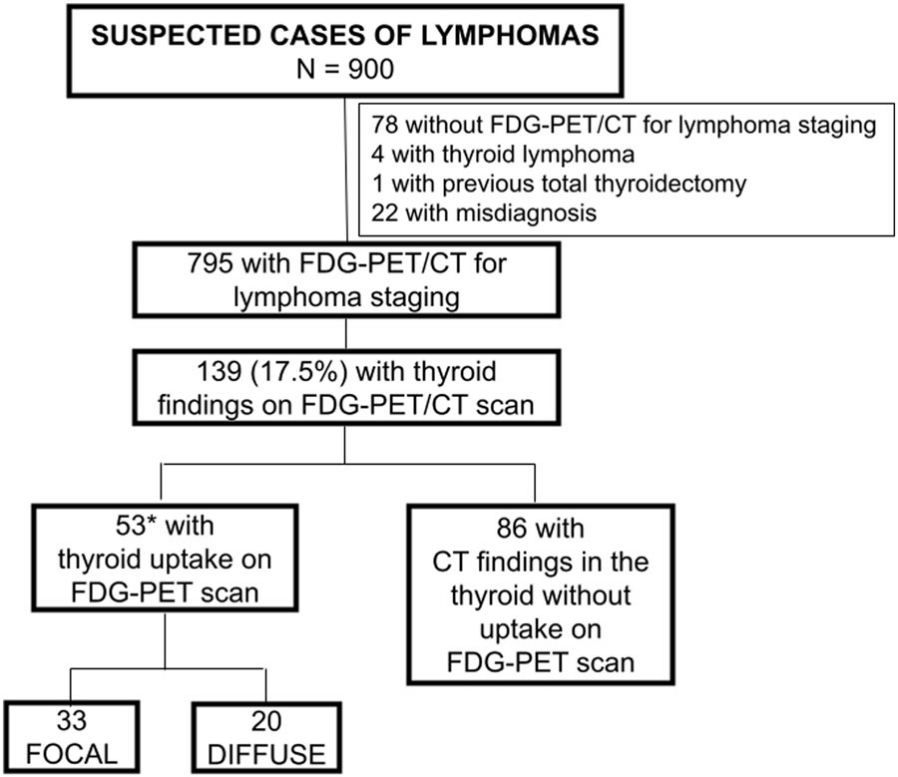
Distribution of thyroid uptake and histological subtypes in lymphoma patients. CT, computed tomography; FDG‐PET, positron emission tomography with 18F‐fluorodeoxyglucose; FDG‐PET/CT, positron emission tomography with 18F‐fluorodeoxyglucose combined with computed tomography; *N* = number of patients. * 32 with diffuse large B cell lymphoma, 6 with Hodgkin lymphoma, 6 with follicular lymphoma, 4 with mantle cell lymphoma, 3 with Burkitt lymphoma, 2 with anaplastic large cell lymphoma, one with extranodal T‐/NK‐cell lymphoma, large B cell and anaplastic large cell lymphomas.

Thyroid abnormalities were observed in 139 patients (17.5%). Among these, 53 patients (6.7%) exhibited FDG‐PET uptake—33 (62.3%) with focal uptake and 20 (37.7%) with diffuse uptake—and 86 (10.8%) had abnormalities detected only on CT (Figure 1).

### 3.1 Demographic Differences

Patients with thyroid uptake were significantly older than those without uptake (61.3 ± 15.8 years vs. 48.9 ± 18.1 years, *p* < 0.01). Female patients were more likely to exhibit thyroid uptake (63.5% vs. 43.8%, *p*  =  0.01). Logistic regression confirmed age and female gender as independent predictors of thyroid uptake, with each additional year of age increasing the odds by 4.1% (OR  =  1.04; 95% CI: 1.02–1.06; *p*  <  0.01) and female gender doubling the odds (OR  =  2.16; 95% CI: 1.20–3.90; *p*  =  0.01).

**Figure 2:**
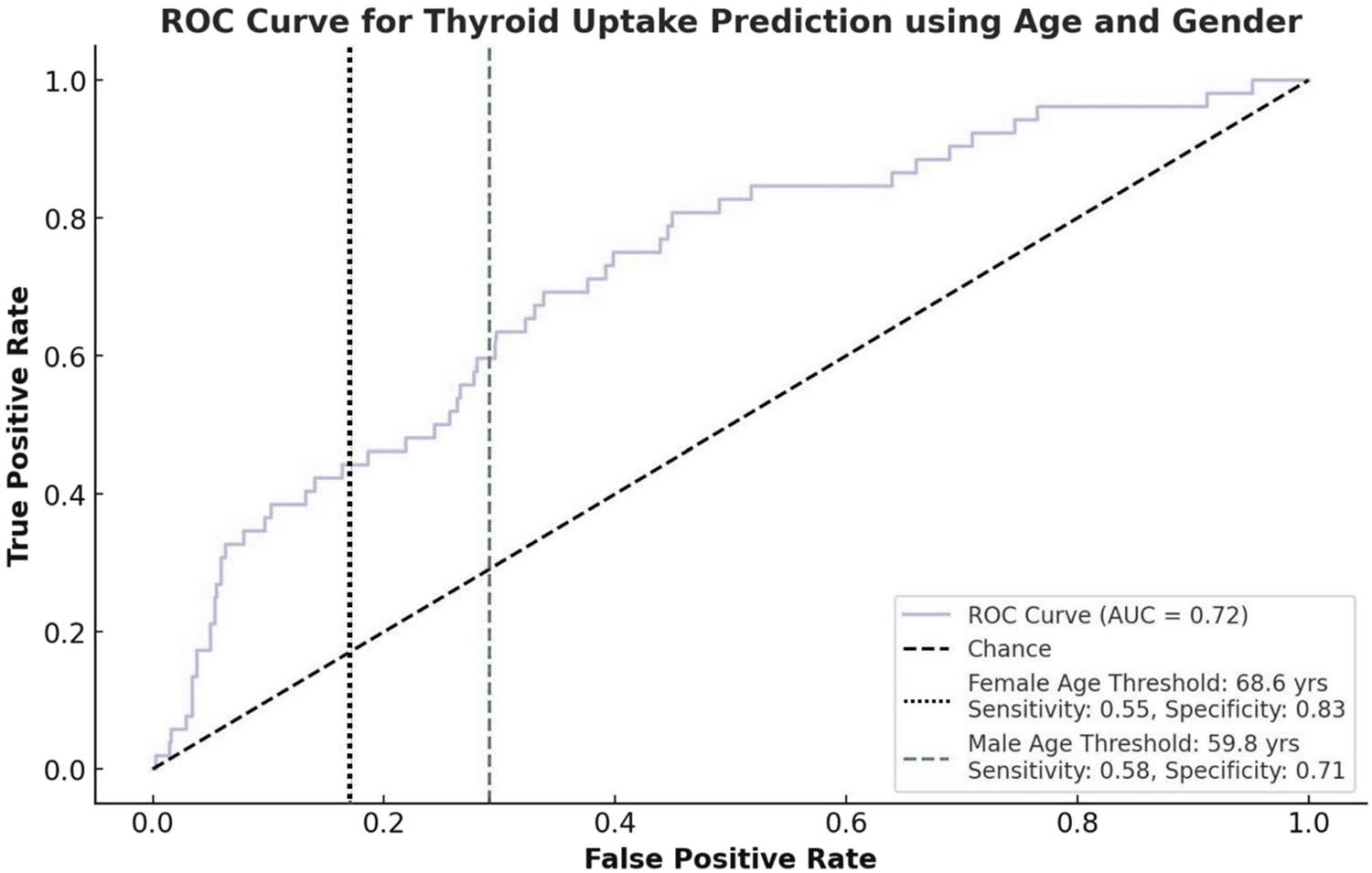
ROC curve for predicting thyroid uptake on FDG‐PET/CT by age and gender in lymphoma patients. Legend: The ROC curve for predicting thyroid uptake using both age and gender as predictors yielded an AUC of 0.72, indicating moderate discrimination. The optimal age threshold for predicting thyroid uptake was 68.6 years for females and 59.8 years for males, highlighting the role of age and gender in assessing incidental thyroid uptake risk during lymphoma staging.

ROC analysis (Figure 2) yielded an AUC of 0.72 for predicting thyroid uptake using age and gender. Optimal age thresholds were determined to be 68.6 years for females and 59.8 years for males, suggesting that older age is a stronger predictor among females.

### 3.2 Thyroid Uptake Patterns and Malignancy Risk

**Figure 3:**
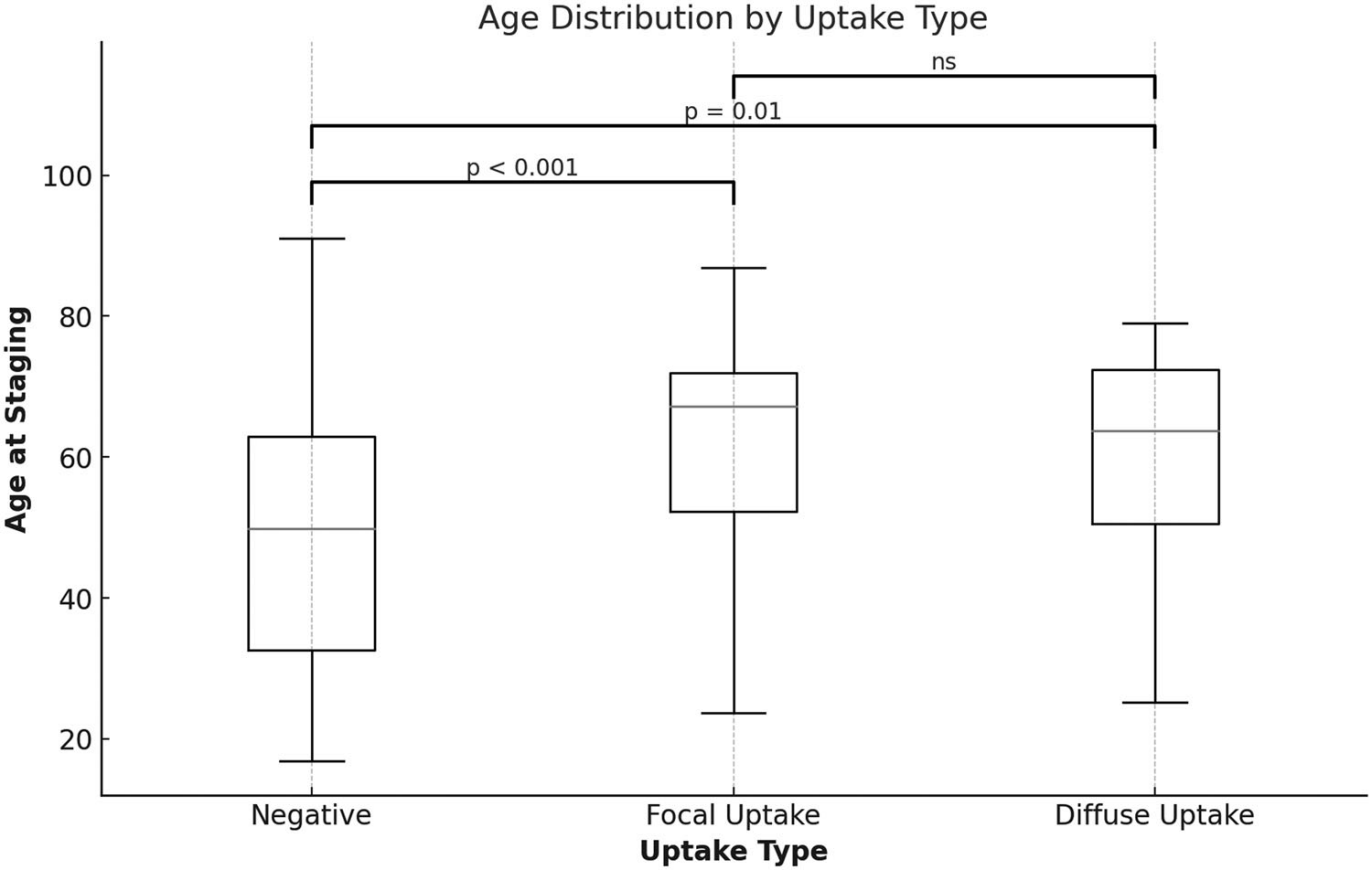
Age distribution by FDG‐PET/CT thyroid uptake type in lymphoma patients. Legend: Box plot illustrating age distribution at staging among lymphoma patients, stratified by thyroid FDG uptake type (Negative, Focal, and Diffuse). Median age, interquartile range (IQR) and full age range are displayed.

The median age of patients without thyroid uptake was 49.8 years (32.6–62.9), compared to 67.3 years (52.2–71.9) and 63.7 years (50.5–72.4) in the focal and diffuse uptake groups, respectively. Both focal and diffuse uptake groups were significantly older than the no‐uptake group (*p* < 0.01 and *p* = 0.01, respectively), with no significant difference between focal and diffuse groups (*p* = 0.74) (Figure 3).

Among the 33 patients with focal FDG uptake, 23 underwent thyroid ultrasound. The maximum nodule diameter ranged from 4 mm to 41 mm, with a median size of 18 mm (interquartile range: 13–27 mm). ACR TI‐RADS classifications were as follows: two nodules were classified as ACR TI‐RADS 1, six as ACR TI‐RADS 3, nine as ACR TI‐RADS 4, and six as ACR TI‐RADS 5. Twenty‐one patients underwent US‐guided FNAC, with Bethesda classifications distributed as follows: one case was Bethesda I (repeat FNAC was not feasible), nine were Bethesda II, three Bethesda III, three Bethesda IV, two Bethesda V, and three Bethesda VI.

A total of 16 patients had SUVmax values greater than 5.0. Among them, 12 patients underwent FNAC: five had Bethesda III or IV cytology with oncocytic features, with histopathological findings of oncocytic hyperplasia, oncocytic adenoma, or the oncocytic subtype of papillary thyroid carcinoma; five had Bethesda II cytology; one had Bethesda VI cytology but passed away before thyroidectomy; and one had Bethesda I cytology, but was unable to repeat FNAC. One additional patient had hyperthyroidism without cytological evaluation.

Bethesda classification showed a strong and statistically significant positive correlation with malignancy (*ρ*  =  0.87, *p* < 0.001). In contrast, no significant correlations were found between malignancy and SUVmax (*ρ* =  –0.17, *p* = 0.53), ACR TI‐RADS classification (*ρ* =  0.18, *p* = 0.50), or Hounsfield unit values (ρ  =  –0.14, *p* = 0.60). Nodule volume on CT demonstrated a moderate negative correlation with malignancy (*ρ* =  –0.50, *p* = 0.046). Furthermore, SUVmax did not significantly correlate with either ACR TI‐RADS classification (*ρ* = 0.29, *p* = 0.20) or Bethesda category (*ρ* =  –0.04, *p* = 0.86), suggesting that metabolic activity measured by FDG uptake was not predictive of sonographic or cytological features.

All malignant cases occurred in nodules classified as ACR TI‐RADS category 4 or 5. Although this trend was notable, the association did not reach statistical significance (*p* = 0.115, Fisher's exact test), likely due to the small sample size.

Among patients with focal thyroid uptake, no significant association was observed between the presence of calcifications on CT and malignancy. Calcifications were observed on CT in three cases, only one of which was malignant. Fisher's exact test indicated no statistically significant association (*p* = 1.00), suggesting that CT‐detected calcifications alone were not predictive of malignancy in this cohort.

ROC curve analysis for predicting malignancy yielded AUC values of 0.45 for SUVmax, 0.42 for Hounsfield units, and 0.19 for CT‐based nodule volume. The AUC for ACR TI‐RADS classification was 0.65, suggesting modest discriminatory ability in this cohort.

Six cases (28.6% of those who underwent FNAC and 18.2% of all patients with focal FDG uptake) were diagnosed with PTC. Among patients with thyroid cancer, SUVmax values ranged from 2.27 to 14.95, with a median of 4.61 (interquartile range: 3.07–7.4). On ultrasound, these malignant nodules measured between 12 mm and 22 mm in maximum diameter, with a median size of 15 mm (interquartile range: 12–18 mm). Five cases were classified as ACR TI‐RADS category 4 and one case as category 5. Cytological analysis revealed Bethesda category V or VI in five cases, and one case was classified as Bethesda IV. One patient was deemed unfit for surgical intervention due to clinical status. The patient with Bethesda IV cytology underwent thyroidectomy, with histopathology confirming the oncocytic subtype of PTC; this case demonstrated focal FDG uptake with a SUVmax of 7.4. The remaining four patients who underwent surgery had histopathological confirmation of the classical subtype of PTC. Notably, four of the six patients diagnosed with PTC had SUVmax values below 5.0.

Among patients with focal thyroid uptake and confirmed histological or cytological confirmation of benign or malignant lesions, CT volumetric and density (HU) were available for analysis. Benign lesions exhibited significantly greater volume than malignant ones (median volume: 3.95 cm³ vs. 1.61 cm³; *p* = 0.039). No significant difference was found in Hounsfield unit values between benign and malignant groups (median HU: 43.5 vs. 28.3; *p* = 0.65).

Among the 20 patients with diffuse thyroid uptake, 58.3% had positive thyroid autoantibodies, a significantly higher rate than in the focal uptake group (5.3%, *p*  <  0.01), indicating an association with autoimmune thyroiditis.

Among the 86 patients without thyroid uptake and thyroid findings on CT, 23 patients had thyroid nodules. Of these, 13 underwent thyroid ultrasound, with ACR‐TIRADS classifications as follows: three ACR‐TIRADS 1, three ACR‐TIRADS 2, four ACR‐TIRADS 3, two ACR‐TIRADS 4, and one ACR‐TIRADS 5. Five patients underwent US‐guided FNAC, and all cases were classified as Bethesda II, with no thyroid carcinoma detected.

### 3.3 Lymphoma Subtype and Thyroid Uptake

NHL patients were older than HL patients (55.3 ±  16.7 vs. 37.7 ±  15.0 years, *p*  <  0.01), although gender distribution did not differ significantly. Diffuse thyroid uptake was more frequent among NHL patients (*p*  < 0.01); however, after adjusting for age, lymphoma subtype was not a significant predictor of thyroid uptake patterns (OR = 1.02; 95% CI: 0.65–1.60; *p*  = 0.93) or SUVmax values (coefficient = 4.01, *p*  = 0.22).

### 3.4 Survival Analysis

The mean follow‐up time was 3.11 ± 2.00 years, with no significant difference based on thyroid uptake (3.10 ± 1.99 years for no uptake vs. 3.19 ± 2.12 years for any uptake, *p* = 0.77). Among patients with no thyroid uptake, 466 (62.7%) achieved cure, compared to 35 (67.3%) among those with thyroid uptake (*p* = 0.61). Regarding mortality, 215 deaths (28.9%) occurred in the no‐uptake group compared to 13 deaths (25.0%) in the uptake group (*p* = 0.65).

**Figure 4:**
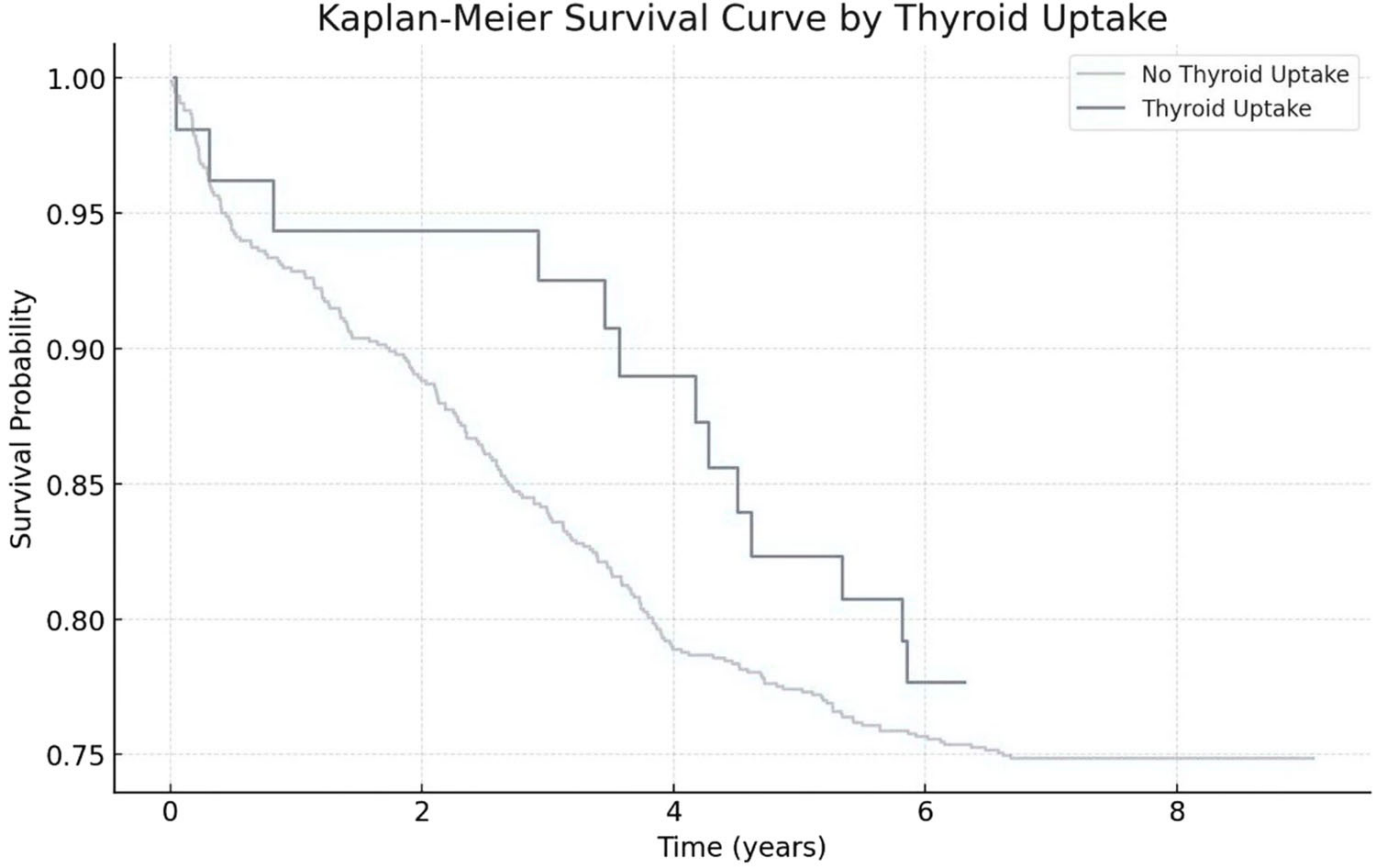
Kaplan‐Meier survival curves comparing overall survival probabilities between patients with and without thyroid uptake. Legend: Survival curves demonstrate overlapping probabilities, with no significant difference observed between the groups (log‐rank test, *p* = 0.65).

Kaplan‐Meier analysis revealed no significant difference in overall survival between patients with and without thyroid uptake (log‐rank test, *p* =  0.65), as illustrated in Figure 4. Cox regression analysis confirmed that thyroid uptake was not an independent predictor of mortality when adjusting for age, gender, and lymphoma subtype.

## 4 Discussion

The detection of thyroid incidentalomas during lymphoma staging presents unique challenges and opportunities for endocrine evaluation. In this study, thyroid abnormalities were identified in 17.5% of patients undergoing FDG‐PET/CT, a higher prevalence than previously reported in lymphoma cohorts [9]. This may reflect differences in imaging protocols, population characteristics, or inclusion criteria. Older age and female gender were independent predictors of thyroid uptake, consistent with epidemiological trends of thyroid disease [5, 6, 7, 12]. ROC curve analysis provided clinically relevant age thresholds, which could assist clinicians in identifying patients at increased risk of incidental thyroid findings.

Prior studies have reported malignancy rates ranging from 9% to 27% in focal incidentalomas detected on FDG‐PET performed for various types of cancer [5, 6, 7] and up to 30% in a cohort of patients with lymphoma ^9^. In our study, the malignancy rate was 18.2%, and 28.6% among those who underwent FNAC. This slightly lower rate may be attributed to stricter histopathological confirmation criteria, variation in the application of Bethesda classification thresholds for FNAC indication, or the high proportion of oncocytic lesions (41.7%) in our cohort, which are known to demonstrate FDG avidity despite their benign nature [8, 13].

Oncocytic (formerly Hürthle cell) adenomas are characterised by high mitochondrial content and glycolytic activity, leading to intense FDG uptake and potential misinterpretation as malignant lesions on FDG‐PET [13]. These nodules often result in indeterminate cytology (Bethesda III or IV). Scappaticcio et al. [14] highlighted the overrepresentation of oncocytic lesions among indeterminate nodules in their meta‐analysis, which is consistent with our findings.

In our analysis, SUVmax alone did not reliably differentiate benign from malignant nodules. No significant correlations were found between SUVmax and either ACR TI‐RADS or Bethesda classification. ROC curve analysis yielded an AUC of 0.45, with limited sensitivity (16.7%) despite high specificity (92.9%) at the optimal threshold (SUVmax = 14.95). These findings are in line with those of Haydardedeoglu et al [15]. Although Nasr et al. [16] reported higher SUVmax and HU values in malignant nodules, methodological differences in CT acquisition and lesion characteristics may explain the discrepancy.

In our cohort, benign lesions exhibited significantly greater volume than malignant ones, while Hounsfield unit values did not differ significantly. This suggests that, although CT‐based volume may provide supplementary information, neither metabolic nor morphological FDG‐PET/CT parameters should be interpreted in isolation. Ultrasound and, when indicated, FNAC remain critical for the evaluation of incidentalomas [17].

These findings highlight the importance of further evaluation with ultrasound and, when indicated, US guided‐FNAC, as recommended by the American Thyroid Association guidelines [17].

Notably, four of the six papillary thyroid carcinoma (PTC) cases in our cohort had SUVmax values below 5.0, and all measured ≤ 22 mm. These findings support the potential role of FDG‐PET/CT in the incidental early detection of small, subclinical thyroid carcinomas in lymphoma patients. While FDG uptake is not specific for malignancy, it may reveal clinically silent lesions requiring endocrine referral.

All malignancies occurred in nodules classified as ACR TI‐RADS 4 or 5. However, the association between the ACR‐TI‐RADS category and malignancy did not reach statistical significance, likely due to small sample size. Still, ROC curve analysis yielded an AUC of 0.65 for ACR‐TI‐RADS, suggesting modest discriminatory performance.

As expected, Bethesda classification was strongly correlated with malignancy (*ρ* = 0.87, *p* < 0.001), reaffirming its value in cytological risk stratification. In contrast, only a moderate inverse correlation was observed between nodule volume and malignancy (*ρ* = –0.50, *p* = 0.046), indicating that smaller nodules were more frequently malignant in this cohort.

Diffuse uptake was significantly associated with positive thyroid autoantibodies (58.3%), supporting the diagnosis of autoimmune thyroiditis—a condition commonly encountered in lymphoma patients due to underlying immune dysregulation [6, 8, 10]. This reinforces the utility of thyroid autoantibody testing and thyroid function monitoring in the evaluation of diffuse uptake [10, 18].

**Figure 5:**
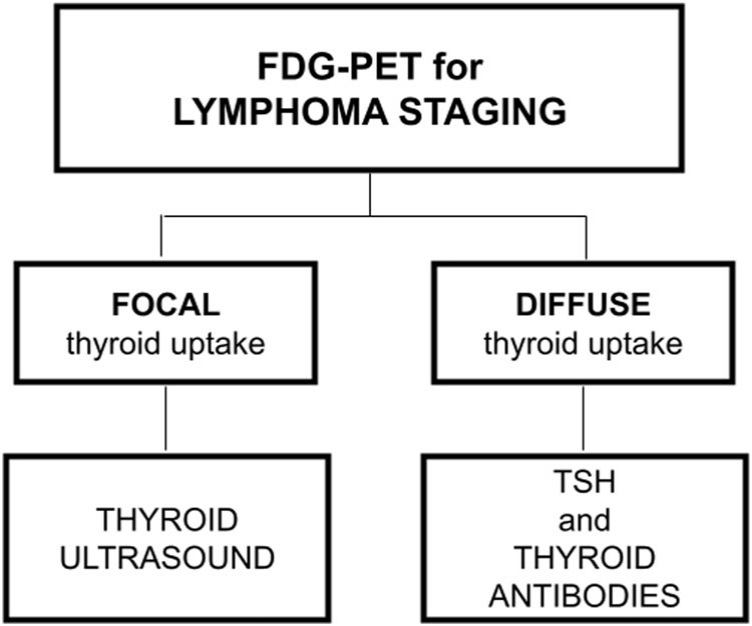
Recommended algorithm for thyroid assessment during lymphoma staging using FDG‐PET. The algorithm distinguishes between focal and diffuse thyroid uptake patterns, guiding subsequent evaluations with ultrasound, TSH measurement, and thyroid autoantibody testing. FDG‐PET, positron emission tomography with 18 F fluorodeoxyglucose; TSH, thyroid‐stimulating hormone.

Figure 5 presents a practical algorithm, derived from our findings, to guide the evaluation of incidental thyroid uptake during lymphoma staging, distinguishing focal from diffuse uptake and outlining appropriate follow‐up investigations.

Importantly, incidental thyroid uptake did not significantly impact overall survival or lymphoma‐specific outcomes. This suggests that, while endocrine evaluation is warranted, thyroid findings should not prompt alteration of oncological treatment protocols. Nevertheless, timely evaluation of focal uptake remains crucial to avoid delayed diagnosis of thyroid cancer.

This study has limitations, including its retrospective design, single‐centre nature, and incomplete data for certain variables (e.g. thyroid function tests and autoantibody levels). The number of patients with complete cytological, histopathological, and imaging data was limited, affecting statistical power for some analyses. However, the study provides robust insight into the metabolic and clinical profile of thyroid incidentalomas in a large, well‐characterised cohort of lymphoma patients.

Prospective multicentre studies are warranted to confirm these findings, assess the generalisability of FDG‐PET/CT‐based risk models, and refine strategies for integrating metabolic, anatomical, and serological parameters into the evaluation of thyroid incidentalomas in this population.

## 5 Conclusion

Thyroid incidentalomas are common among lymphoma patients undergoing FDG‐PET/CT, particularly older women. Focal thyroid uptake carries a higher risk of malignancy and should prompt further investigation, even when SUVmax values are low. Diffuse uptake is strongly associated with thyroid autoimmunity, highlighting the importance of thyroid function monitoring. Clinicians should remain vigilant when interpreting thyroid findings on FDG‐PET/CT to ensure appropriate endocrine management without delaying oncological care.

## Disclosure

The authors report there are no competing interests to declare.
